# Intimate partner violence against women in rural Vietnam - different socio-demographic factors are associated with different forms of violence: Need for new intervention guidelines?

**DOI:** 10.1186/1471-2458-8-55

**Published:** 2008-02-11

**Authors:** Nguyen Dang Vung, Per-Olof Ostergren, Gunilla Krantz

**Affiliations:** 1 Department of Demography, Faculty of Public Health, Hanoi Medical University, Hanoi, Vietnam; 2 Division of Social Medicine and Global Health, Department of Health Sciences, Lund University, Sweden; 3 Department of Community Medicine and Public Health, Sahlgrenska Academy at Goteborg University, Goteborg, Sweden; 4 Division of International Health, Ihcar, Department of Public Health Sciences, Karolinska Institutet, Stockholm, Sweden

## Abstract

**Background:**

This population-based study investigated the different forms, magnitude and risk factors of men's violence against women in intimate relationships in a rural part of northern Vietnam and whether a difference in risk factors were at hand for the different forms of violence. Vietnam has undergone a rapid transition in the last 20 years, moving towards a more equal situation for men and women however, Confucian doctrine is still strong and little is known about men's violence against women within the Vietnamese family.

**Methods:**

This is a cross-sectional population-based study that used a questionnaire developed by the World Health Organisation for investigating women's health and violence against women in different settings. Face-to face structured interviewing was performed and 883 married women, aged 17 to 60 participated. Bi- and multivariate analyses was used for risk factor assessment.

**Results:**

The lifetime prevalence of physical violence was 30.9 percent and past year prevalence was 8.3 per cent, while the corresponding figures for physical and sexual violence combined was 32.7 and 9.2 percent. The lifetime prevalence was highest for psychological abuse (27.9 percent) as a single entity. In most cases the violence was of a severe nature and exercised as repeated acts over time. Woman's low educational level, husband's low education, low household income and the husband having more than one wife/partner were risk factors for lifetime and past year physical/sexual violence. The pattern of factors associated with psychological abuse alone was however different. Husband's low professional status and women's intermediate level of education appeared as risk factors.

**Conclusion:**

Men's violence against women in intimate relationships is commonly occurring in rural Vietnam. There is an obvious need of preventive and treatment activities. Our findings point at that pure psychological abuse is different from physical/sexual violence in terms of differing characteristics of the perpetrators and it might be that also different strategies are needed to reduce and prevent this violence.

## Background

An increasing number of studies on violence against women performed in high and low income countries since the late 1980's [1,2] report on prevalence and risk factors of physical, psychological and sexual abuse but few studies, mainly from the USA, investigated how the different forms relate to each other [3,4]. Further, there are no population based studies of magnitude from Vietnam.

The issue of intimate partner violence has this far received limited attention from the legislature, the judiciary and governmental institutions in Vietnam, while media has paid it attention in the past years. In 2002 however, IPV was officially recognised as an obstacle to development and to reduce men's violence against women has now been included in Vietnam's Millennium development targets [5].

Vietnam has undergone a rapid transition in the last 20 years, moving from a planned economy system to a market economy and towards a more equal situation for men and women. This change process, referred to as 'renovation' (Doi Moi), holds promotion of the private sector, implementation of state enterprise and agrarian reforms, and women's rights have been underscored [6,7]. However, strong cultural traditions, often centred on patriarchal norms about family and traditional gender norms, continue to prevail despite being increasingly in disagreement with the economic reality men and women face [5]. Today, more than 70 percent of women of working age (16–55) participate in the labour market and women constitute 52 percent of the total work force [8]. Despite this, males generally hold a dominant position within and outside the household and continue to be seen as the primary breadwinners while women assume primary responsibility for housework and childcare and are expected to maintain "family happiness and harmony" [9]. Current gender relations are a compound of norms, values and practices influenced by traditional Confucian doctrine advocating patriarchal norms and as well by more recent socialist ideologies together with changes associated with the current period of transition and integration into the global economy [10].

Polygamy is still practiced in rural areas although it became illegal in 1960. It has been justified on the grounds that the family in Vietnam's traditional patriarchal society formed the main economic unit, where women performed the main bulk of work but under male supervision. Consequently, the more wives, daughters and female servants a male could have, the more work could be performed and the more the family could produce [11]. Official documents (Gender equity and the marriage and family law) state that polygamy is today virtually non-existent apart from in some rural areas where the law is difficult to apply. The actual number of polygamous relationships is not officially known.

Only a limited number of studies have so far been performed on IPV in Vietnam and these mainly used a qualitative research approach. Small-scale studies reveal that IPV occurs in urban and rural settings and in all social strata [12,13]. Another survey found that an estimated 60 percent of divorce cases were the result of physical maltreatment of the woman by the husband [14]. In one of our qualitative studies, it was brought to our attention that perpetrators of physical violence possibly differ in terms of educational attainment and income to perpetrators of psychological abuse [15].

Considering the above, the objective of this population based study was to investigate intimate partner violence directed at women in rural Vietnam, by in detail delineating the different forms of violence, their occurrence and overlapping, focusing also on the severity of the violence and to test the hypothesis that the different forms of violence might carry different socio-demographic and psychosocial risk factors.

## Methods

### Definitions

*Intimate partner violence *(IPV) is defined by WHO as any acts of physical, sexual and emotional abuse by a current or former partner whether cohabiting or not [2]. Other terms used in the scientific literature are domestic violence, battering or wife abuse [16].

### Design and sample

This study was conducted in 2002 within the framework of the demographic surveillance site in Bavi District, Ha Tay Province, called FilaBavi, situated in northern rural Vietnam [17].

In Bavi district, agricultural production and livestock breeding are the main economic activities of the local people (81%) [17]. Illiteracy is low (0.4%), but higher among women than among men. About 70% of the adult population has completed primary school, 21% secondary level, 9% high school and 0.6% higher education. The higher the educational level, the lower is the number of females [18].

FilaBavi consists of a cohort of approx. 50.000 individuals (69 clusters) set together through a stratified cluster sampling procedure from the 240.000 individuals living in the district. For this study, 37 clusters were selected through a random cluster sampling technique. A number of households were selected from each cluster, proportional to the total number of households in each cluster. Eligible for the study were married or partnered women aged 17 to 60 years.

Face-to-face interviewing was used for data collection. The 39 female interviewers and six field supervisors engaged in the regular FilaBavi data collections were trained by the principal investigator in how to manage the specific challenges and difficulties encountered in studies on violence. As IPV might generate feelings of insecurity and frustration also among the interviewers, a pilot study was performed and the interviewers were encouraged to renounce participation if not feeling comfortable, but no one did.

Based on power calculations to detect stable significant risks of IPV, a total of 884 households containing a married or partnered woman were randomly selected for participation. Of these, 867 were currently married, and 16 were in a stable sexual relationship with a man, but not married. Only one woman declined to participate due to psychiatric illness. The participating 883 women all completed the interview and are henceforth referred to as married women.

### Measurements

The data collection instrument used was the Multi-country Study on Women's Health and Life Experiences Questionnaire developed by the World Health Organization (WHO) for studies within public health with focus on interpersonal violence [19]. The questionnaire was developed for use in different cultures and is considered to be cross-culturally appropriate. The abuse questions were developed on the basis of a variety of other abuse assessment scales (Index of Spouse Abuse and the Conflict Tactics Scales) with established reliability and construct validity [20,21]. This instrument was revised and translated into Vietnamese. The revisions made consisted of selected sections and items being removed as this data was either obtained from the Filabavi database (socio-demographic data) [11] or considered inappropriate in the Vietnamese context (dowry related items). In a one-day seminar and a pilot interview, the questionnaire was further validated through a review panel process where each item was considered for appropriateness.

Only women took direct part in this study and data related to husbands/partners were obtained from the participating women.

Violence occurrence was assessed by types (physical, psychological and sexual abuse), timing (life-time and past year exposure) and frequency (how often it occurred). Physical abuse was assessed by 11 items: slapping, throwing things, pushing or shoving which were classified as moderate physical abuse behaviours. Further hitting, kicking, dragging, beating, choking, burning and threatening with or using a weapon (knife, scissors or object) were classified as severe physical violence [19]. Sexual abuse was assessed by three items: having sexual intercourse against the respondent's will, using physical force for sexual intercourse, and forcing the respondent to sexually degrading acts. Psychological abuse was assessed by four items: insults or degrading activities, belittlement or humiliation, scaring the respondent on purpose including threats of violence.

Two dependent variables were created, physical and sexual violence combined and pure psychological abuse. Physical and sexual violence was defined as the respondent being subjected to any act of physical or sexual violence or both (henceforth referred to as physical/sexual violence); psychological abuse was defined as being subjected to any item of psychological abuse without overlap of any other kind of violence (referred to as psychological abuse alone) [22].

Lifetime occurrence of any kind of violence was defined as experience of any act of violence to date of the interview from a current or former husband/partner. Abuse taking place within the past year was defined as any act taking place within the past 12 months. For bi- and multivariate analyses the dependent variables were dichotomised into experience of violence as opposed to no experience of violence. For these analyses, those with only one single experience of violence over the lifetime were considered as non-exposed, to strengthen the criterion for violence exposure.

Socio-demographic and psychosocial variables were tried as independent risk factors. Age was divided into three groups. Educational attainment was grouped into primary (5 years) and secondary schooling (9 years) and higher education (>9 years) respectively, and dichotomised with higher education as the reference category. Annual household income was divided into quintiles and later into three groups (lowest income group, < 288 USD, low and middle income groups, from 288 – 570 USD and high and highest income groups > 570 USD) and further dichotomised for the multivariate analyses whereby a household income in the lowest income group (lowest and low income groups, < 425 USD) was treated as the exposure category. Husband's working specifics was also grouped into three categories and dichotomised into professionals as the reference and semi-skilled and unskilled combined as the exposure group.

### Statistical Analysis

Data were double entered into the Statistical Package for the Social Sciences (SPSS) version 10.0 which was used for all statistical purposes [23]. Risk ratios were estimated by odds ratios (OR). Statistical significance was determined at the 95 percent confidence interval level.

Bi- and multivariate analyses were adjusted for age apart from when age differences were investigated. For the multivariate analyses, variables of theoretical and empirical (statistically significant in the bivariate analyses) interest were entered one by one in a stepwise fashion. To avoid a correlation effect, the multiple logistic regression models included only items with correlation coefficients below 0.4 [24].

### Ethical considerations

The World Health Organisation has issued guidelines for violence research [25] and these were strictly followed. Interviews were held in strict privacy, mainly in the respondents' homes, with no one able to overhear the conversation. In a few cases when privacy was not possible to establish, the interview was performed at a nearby community health centre. The participants were informed about their possibility to withdraw at any point during the research phase and gave written informed consent to participate. This study was approved by the institutional review board of Gothenburg University, Sweden; Hanoi Medical University and Ministry of Health, Hanoi and Bavi district People's Committee and Bavi District Health Center, Hatay province, Vietnam.

## Results

### Socio-demographic characteristics

Of the participating women, the majority (77.4 percent) were 30 to 60 years of age (Table 1). More women than men had completed secondary school, 63.4 percent and 58.6 percent, respectively, although more husbands had attained a higher level of education. Nearly 90 percent of the respondents were farmers. The majority of the husbands were unskilled workers (73.5 percent) and 15.5 percent (n = 130) of the men had more than one wife/partner. Twenty percent of the study population was extremely poor, and had to stay on a household income below the official poverty line. More than half of the respondents answered to the norm of the two-child policy, i.e. a woman should not give birth to more than two children (55.3 percent) (Table 1).

**Table 1:** Socio-demographic and psychosocial factors of respondents and their husbands. N = 883

**Variables**	**N**	**%**
***Respondents***		

**1. Age groups**		
17–29	200	22.6
30–45	406	46.0
46–60	277	31.4
**2. Education**		
High School & Higher Education (>= 10 years)	134	15.2
Secondary school (6 – 9 years)	560	63.4
Primary School (< 6 years)	189	21.4
**3. Occupation**		
Agriculture labour	761	86.2
Other (hired labour, breeding farmer, tailors, construction assistants, etc...)	122	13.8

***Husbands/Partners***		

**4. Age groups**		
20–29	108	12.5
30–45	442	51.0
46–77	316	36.5
**5. Education**		
High School & Higher Education (>= 10 years)	180	22.0
Secondary school (6–9 years)	481	58.6
Primary School (< 6 years)	159	19.4
**6. Working specifics**		
Professional	171	22.0
Semi-skilled	35	4.5
Unskilled	573	73.5
**7. Husbands having more than one wife/partner**		
No	710	84.5
Yes	130	15.5

***Households***		

**8. HH Income per year**		
< 288 USD	176	20.0
288 – 570 USD	353	40.1
> 570 USD	351	39.9
**9. Number of children**		
No children	16	1.8
(From) 1–2 children	466	53.5
(From) 3 children and more	389	44.7

### Forms of violence

Of the 883 women, 30.9 percent (n = 273) had been subjected to any form of physical violence in their lifetime, and 8.5 percent in the preceding year (Table 2) and for the combined exposure to physical and sexual violence, the corresponding figures were 32.7 and 9.2 percent. The most commonly occurring form was psychological abuse (lifetime 55.4 percent, n = 489; past year 33.7 percent, n = 298). For physical violence reported over the lifetime, 47 percent was classified as being severe and for the past year it was 53 percent. In the majority of cases, the violence was exerted as repeated acts. Lifetime experience of sexual violence was reported by 6.6 percent of the women, and by 2.2 percent for previous year exposure.

**Table 2:** Lifetime and past year prevalence of different forms of violence among married women. N = 883

	**Lifetime prevalence**				**Past year prevalence**			
**Forms of Violence**	**Violence exp. % (n)**	**Number of events**			**Violence exp. % (n)**	**Number of events**		
		**Once**	**2–5 times**	**> 5 times**		**Once**	**2–5 times**	**> 5 times**
**Physical Violence §**		% (n)	% (n)	% (n)		% (n)	% (n)	% (n)
*Moderate physical violence:*	16.3(144)	5.9 (52)	8.2 (72)	2.3 (20)	3.9 (34)	2.6 (23)	1.0 (9)	0.2 (2)
- Slapped/threw something	27.0 (238)	6.7 (59)	12.1 (107)	8.1 (72)	7.1 (63)	3.3 (29)	3.1 (27)	0.8 (7)
- Pushed/Showed	5.8 (51)	0.8 (7)	1.9 (17)	3.1 (27)	2.6 (23)	0.7 (6)	1.4 (12)	0.6 (5)
*Severe physical violence:*	14.6(129)	2.9 (26)	6.1 (54)	5.5 (49)	4.4 (39)	0.8 (7)	2.8 (25)	0.8 (7)
- Hit that could hurt	11.6 (102)	2.6 (23)	3.7 (33)	5.2 (46)	3.4 (30)	1.1 (10)	1.6 (14)	0.7 (6)
- Kicked/dragged or beating	8.6 (76)	1.9 (17)	3.1 (27)	3.6 (32)	3.1(27)	1.2 (11)	1.2 (11)	0.6 (5)
- Choked or Burnt	1.7 (15)	0.6 (5)	0.6 (5)	0.6 (5)	0.3 (3)	0.0 (0)	0.2 (2)	0.1 (1)
- Threaten or Used a weapon	1.8 (16)	0.8 (7)	0.5 (4)	0.6 (5)	0.5 (4)	0.1 (1)	0.2 (2)	0.1 (1)
***Summary measure of Physical Violence***	**30.9 (273)**	**8.5 (75)**	**22.4 (198)**		**8.3 (73)**	**3.6 (32)**	**4.6 (41)**	
**Sexual Violence §**								
- Physically forced to have sexual intercourse	2.7 (24)	0.2 (2)	1.1 (10)	1.4 (12)	0.5 (4)	0.0 (0)	0.2 (2)	0.2 (2)
- Did not want to have sex. Intercourse	4.9 (43)	0.7 (6)	2.4 (21)	1.8 (16)	1.8 (16)	0.9 (8)	0.7 (6)	0.2 (2)
- Forced to do something sexual that felt degrading	1.0 (9)	0.0 (0)	0.6 (5)	0.5 (4)	0.5 (4)	0.0 (0)	0.2 (2)	0.2 (2)
***Summary measure of Sexual Violence***	**6.6 (58)**	**0.7 (6)**	**5.9 (52)**		**2.2 (19)**	**0.9 (8)**	**1.3 (11)**	
**Physical and sexual violence**	**32.7 (289)**	**10.0 (88)**	**22.8 (201)**		**9.2 (81)**	**4.1 (36)**	**5.1 (45)**	
**Psychological abuse §**								
- Insulted or made her feel bad about herself	20.0 (177)	0.9 (8)	6.1 (54)	12.6 (111)	9.4 (83)	1.4 (12)	5.2 (46)	2.8 (25)
- Belittled or humiliated her	10.6 (94)	0.2 (2)	3.5 (31)	6.7 (59)	5.7 (50)	1.2 (11)	3.3 (29)	1.1 (10)
- Did things to scare or intimidate her on purpose	49.2 (434)	1.9 (17)	13.0 (115)	32.5 (287)	30.4 (268)	5.9 (52)	18.7 (165)	5.8 (51)
- Threaten to hurt her or someone she cared about	12.9 (114)	1.0 (9)	5.3 (47)	6.3 (56)	5.9 (52)	1.2 (11)	3.2 (28)	1.5 (13)
***Summary measure of Psychological abuse***	**55.4 (489)**	**2.2 (19)**	**53.2 (470)**		**33.7 (298)**	**5.3 (47)**	**28.4 (251)**	
**Psychological abuse alone**	**27.9 (246)**	**1.8 (16)**	**26.0 (230)**		**25.4 (224)**	**4.3 (38)**	**22.4 (186)**	
**Summary measures all forms of violence**	**60.6 (535)**	**10.0 (88)**	**50.6 (447)**		**34.5 (305)**	**5.4 (48)**	**29.8 (263)**	

§ Can occur more than once

**Figure 1:**
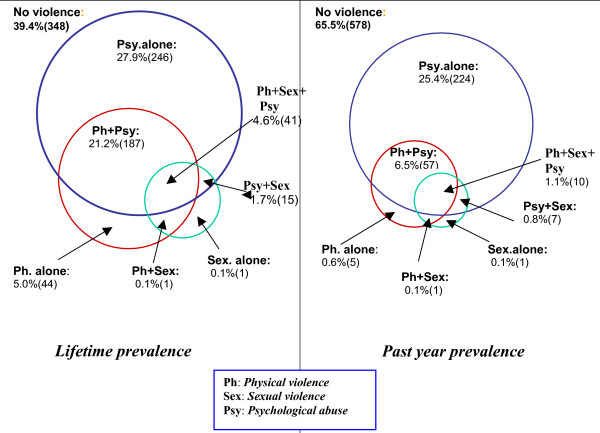
Overlaps between different types of intimate partner violence. N = 883 married women.

The different forms of violence and their overlapping are displayed in detail in the Venn diagrams in Figure 1. These illustrate that the most commonly occurring form of violence was psychological abuse alone (lifetime 27.9 percent, n = 246; past year 25.4 percent, n = 224) followed by physical and psychological abuse combined (lifetime 21.2 percent, n = 187; past year 6.5 percent, n = 57). Just one woman reported sexual violence as a single exposure (0.1 percent).

The two dependent variables selected for further analyses were physical and sexual violence combined (lifetime 32.7 percent, n = 289; past year 9.2 percent, n = 81) and psychological abuse alone.

### Associations with socio-demographic and psychosocial factors

The youngest age group was the most exposed to physical/sexual and also psychological abuse alone in the past year, with a slight decrease over the age groups. The number of children in the family did not influence violence occurrence, irrespective of type of violence or time span (Table 3 and 4).

**Table 3:** Associations between socio-demographic and psychosocial variables and lifetime and past year physical/sexual violence, age adjusted. N = 883

**Variables**	**Lifetime prevalence of physical and sexual violence (n = 201)**		**Past year prevalence of physical and sexual violence (n = 81)**	
	**% (n) with violence exp.**	**OR (95%CI)**	**% (n) with violence exp.**	**OR (95%CI)**

**1. Respondent 's age**				
46–60	23.1 (64)	1	5.1 (14)	1
30–45	23.9 (97)	0.97 (0.49–1.91)	9.6 (39)	1.21 (0.41–3.58)
17–29	20.0 (40)	0.73 (0.24–2.24)	14.0 (28)	1.26 (0.23–6.86)
**2. Respondent's Education**				
High School & Higher Education	12.7 (17)	1	6.7 (9)	1
Secondary school	24.6 (138)	2.25 (1.30–3.87)	10.2 (57)	1.67 (0.80–3.49)
Primary School	24.3 (46)	2.18 (1.16–4.10)	7.9 (15)	1.89 (0.77–4.63)
**3. Husband's Education**				
High School & Higher Education	14.4 (26)	1	7.2 (13)	1
Secondary school	24.5 (118)	1.93 (1.21–3.06)	8.7 (42)	1.28 (0.67–2.47)
Primary School	28.3 (45)	2.33 (1.35–4.02)	15.1 (24)	2.77 (1.34–5.73)
**4. Annual household Income**				
> 570 USD	16.2 (57)	1	6.0 (21)	1
288 – 570 USD	26.6 (94)	1.91 (1.32–2.77)	10.2 (36)	1.61 (0.91–2.84)
< 288 USD	27.3 (48)	1.98 (1.28–3.07)	13.1 (23)	2.10 (1.12–3.94)
**5. Number of children**				
From 1–2 children	23.4 (109)	1	10.7 (50)	1
From 3 children and more	23.1 (90)	0.96 (0.66–1.40)	7.7 (30)	1.30 (0.72–2.36)
**6. Husband's Working specifics**				
Professional	18.7 (32)	1	7.0 (12)	1
Unskilled & semi-skilled	24.4 (140)	1.43 (0.93–2.21)	10.5 (60)	1.47 (0.77–2.80)
**7. Husbands having more than one**				
**wife/partner**				
No	20.0 (142)	1	9.0 (64)	1
Yes	36.9 (48)	2.44 (1.60–3.72)	8.5 (11)	1.30 (0.64–2.63)

OR: Odds ratios; CI: confidence interval.

**Table 4:** Associations between socio-demographic and psychosocial variables and lifetime and past year psychological abuse alone, age adjusted. N = 883

**Variables**	**Lifetime prevalence of psychological abuse alone (n = 230)**		**Past year prevalence of psychological abuse alone (n = 224)**	
	**% (n) with Violence**	**OR (95%CI)**	**% (n) with Violence**	**OR (95%CI)**

**1. Respondent 's age**				
46–60	26.4 (73)	1	20.6 (57)	1
30–45	25.1 (103	1.32 (0.68–2.53)	26.4 (107)	1.43 (0.73–2.80)
17–29	27.5 (55)	1.96 (0.66–5.78)	30.0 (60)	1.76 (0.59–5.27)
**2. Respondent's Education**				
High School & Higher Education	17.2 (23)	1	18.7 (25)	1
Secondary school	29.5 (165)	2.00 (1.23–3.25)	29.3 (164)	1.83 (1.14–2.94)
Primary School	22.2 (42)	1.29 (0.72–2.33)	18.5 (35)	1.19 (0.63–2.25)
**3. Husband's Education**				
High School & Higher Education	25.6 (46)	1	22.8 (41)	1
Secondary school	28.3 (136)	1.14 (0.77–1.69)	28.1 (135)	1.34 (0.89–2.00)
Primary School	25.2 (40)	0.95 (0.58–1.55)	25.8 (41)	1.24 (0.75–2.04)
**4. Annual household Income**				
> 570 USD	27.4 (96)	1	23.9 (84)	1
288 – 570 USD	26.3 (93)	1.27 (0.83–1.95)	28.0 (99)	1.19 (0.85–1.68)
< 288 USD	22.7 (40)	1.22 (0.80–1.86)	22.2 (39)	0.86 (0.56–1.33)
**5. Number of children**				
From 1-2 children	25.1 (117)	1	26.2 (122)	1
From 3 children and more	28.0 (109)	1.20 (0.84–1.73)	24.4 (95)	0.91 (0.67–1.24)
**6. Husband's Working specifics**				
Professional	14.0 (24)	1	17.0 (29)	1
Unskilled & semi-skilled	30.2 (173)	2.68 (1.68–4.28)	31.2 (179)	2.23 (1.44–3.44)
**7. Husbands having more than one**				
**wife/partner**				
No	27.6 (196)	1	26.9 (191)	1
Yes	21.5 (28)	0.69 (0.44–1.10)	20.0 (26)	0.68 (0.43–1.08)

OR: Odds ratios; CI: confidence interval.

Respondents' as well as partners' low educational attainment were statistically significant risk factors for lifetime physical/sexual violence as was low household income and polygamy (Table 3). If the husband had passed primary school only, the risk of physical/sexual violence was more than twice as high as if the husband was highly educated (OR 2.33 with 95% confidence intervals, CI, of 1.35–4.02). For women married to a husband with multiple wives/partners there was a more than two-fold risk increase of lifetime physical/sexual violence (OR 2.44; 1.60–3.72).

For past year physical/sexual violence, only husband's low educational attainment and the family being extremely poor came out as statistically significant risk factors (OR = 2.77, 1.34–5.73 and OR = 2.10, 1.12–3.94, respectively). Living with a husband with multiple wives/partners failed to reach statistical significance (OR 2.03; 0.87–4.74), probably due to small sample size.

For psychological abuse alone a difference in risk factors appeared. Women with secondary schooling were at twice as high a risk of having experienced violence in her lifetime than those with higher education (OR 2.00; 1.23–3.25) while primary schooling did not prove statistical significance (Table 4). Also, for a woman married to an unskilled/semiskilled worker the risk of psychological abuse was high (OR 2.68; 1.68–4.28), while husbands' educational level was of no statistical significance. The same pattern was found for past year psychological abuse alone.

Interestingly, women married to men with more than one wife/partner were at risk of physical/sexual violence but not of psychological abuse alone. Separate analyses of the 130 polygamous households revealed that they belonged to the poorest strata, that the husbands were likely to be of older age (72 percent were 40 years of age or older), low educated and with son preference attitudes.

In a final step, multiple logistic regression analyses were performed in four separate models with dichotomised independent variables to further investigate chains of associations and to control for possible confounding factors, displayed in Table 5. This procedure did not change the risk factor pattern for either lifetime physical/sexual violence or psychological abuse, whether lifetime or past year exposure. For past year occurrence of physical/sexual violence, only low household income remained as a statistically significant factor all through the analysis (Table 5).

**Table 5:** Associations between socio-demographic and psychosocial variables and physical/sexual and psychological abuse alone, final models, age adjusted. N = 883.

**Variables**	**Physical/sexual violence over the lifetime ** OR (95%CI)	**Physical/sexual violence, past year** OR (95%CI)	**Psychological abuse alone, lifetime** OR (95%CI)	**Psychological abuse alone, past year** OR (95%CI)
Respondent's Education				
Secondary school	1.90 (1.04–3.48)	1.46 (0.65–3.28)	1.86 (1.07–3.23)	1.67 (0.99–2.83)
Primary school	1.69 (0.82–3.47)	1.72 (0.64–4.64)	1.18 (0.59–2.36)	1.17 (0.60–2.28)
Husband's low education	1.77 (1.08–2.92)	1.24 (0.63–2.46)	2.73 (1.66–4.49)	2.41 (1.50–3.87)
Low household Income	1.74 (1.22–2.49)	1.88 (1.13–3.13)	0.80 (0.56–1.16)	0.89 (0.63–1.28)
Husbands with more than one wife/partner	2.48 (1.55–3.98)	1.34 (0.64–2.81)	1.02 (0.60–1.73)	0.93 (0.55–1.59)

OR: Odds ratios; CI: confidence interval.

## Discussion

Intimate partner violence directed at women is commonly occurring in rural Vietnam. Of the women, 31 percent had been physically abused in their lifetime and eight percent in the past year, and the corresponding findings for physical and sexual violence combined was 33 and nine percent. The majority of the women were subjected to psychological abuse, often in combination with severe physical violence repeatedly over time. Our hypothesis that physical/sexual violence might carry different risk factors than psychological abuse found some support.

### Strengths and weaknesses

When researching such a sensitive matter as violence within the family, underreporting is a universal phenomenon [26]. In this study the data was collected by experienced and trained female field interviewers recognised by the respondents as collectors of the general field site data every third month in face-to-face interviews. The field workers were however not living in the same area as the respondents. The fact that the field workers were somewhat known to the respondents could have restrained some women from telling about violence experience, but we believe that this relative familiarity would contribute to feelings of trust and confidence and make disclosure rates higher.

Some of the interviewers might have been exposed to violence themselves. This was not investigated for ethical reasons, but it was discussed in general terms and no one declined to take part in the study although this opportunity was given.

Past year prevalence is often thought to be a more reliable assessment of intimate partner violence because of the assumption of less recall bias [27]. However, recent events of violence might be more difficult to report due to feelings of being ashamed or fear of retaliation when disclosing such family problems, especially sexual violence incidents. We consider both lifetime and past year prevalence useful to report as recall bias ought to be less in studies on such grievous life experiences as violence than when inquiring about less sensitive matters.

It could be argued that this is a case-control study where the cases produced during a certain time period are collected at the same time as the controls. Ideally, exposure of risk factors should be compared between cases and the complete population in a case-control study, while here exposure is compared between cases and non-cases. This will result in an overestimation of risk ratios, if risk factors are more prevalent in the complete population, compared to the non-cases. This is usually an error within acceptable limits if the risk is less than 20 percent [24]. Regarding risk of past year, only 'psychological abuse alone' exceeded this risk (25.4 percent).

The data concerning life-time risk is somewhat more problematic, since cases and controls ideally should be sampled from the same source population. Here, we cannot exclude that cases and controls (non-cases) could represent different source populations since e.g. selection effects and a higher loss of cases than non-cases over time could be the result of mortality and mobility patterns. This will probably tend to underestimate the risks, since one could assume that the loss is higher for cases. Another important bias regarding the life-time risk is of course differential recall bias, but if at hand, it will lead to an underestimation of the found risks. Therefore our results probably represent rather conservative estimates.

### The findings in relation to other studies

From neighbouring China, a study from an out-patient gynaecological clinic used the same violence definitions and methodology as in this study apart from being health-care based [28]. It revealed that 38 percent of the women seeking care reported physical violence experience over the lifetime while 21 percent reported past year exposure and the corresponding figures for physical and sexual violence combined was 43 and 26 percent respectively. In the WHO-multi country study, presenting data from ten countries on lifetime experience of physical violence, severe and repeated violence was reported as ranging from four percent in urban Japan to 49 percent in provincial Peru [29], to be compared with 15 percent in our study. In a population based survey from Nicaragua, the corresponding figure was much higher (52 percent) [30]. These huge variations are due to a number of factors such as differences in definitions of the violence, and in the methodologies used to measure the violence, but also in differences between countries in how willing women are to disclose violence experience and as well in cultural and contextual differences [2]. Disclosing violence experience is also dependent on fear of retaliation and whether any support is available [31].

Sexual violence was probably underreported in our study as less than three percent of the women reported having been physically forced to sexual intercourse. This conclusion is based on findings from one of our earlier qualitative studies where sexual violence was discussed but mainly by health care staff who reported it to be a rather common phenomenon in rural parts [17]. The term "marital rape" appears to be unrecognised in the Vietnamese society however, there is evidence that ''forced sex" in the context of marriage does occur [32] but no cases of marital rape have so far been brought before the Vietnamese court. This is largely due to the perception of conjugal affairs as being private and to the patriarchal norm that wives should obey their husbands and cannot refuse their demands for sex [14].

Few studies investigated the overlaps between different forms of violence but in studies reporting from Nicaragua and South Africa, physical and psychological abuse combined was the most commonly occurring form closely followed by psychological abuse as a single form of violence [30,33], which is opposite from what was found in our study. This might be explained by differences between countries in what acts that are considered as violence, and especially acts of psychological oppression might be interpreted differently in different cultural contexts.

A common finding in many studies is that poor socio-economic conditions contribute to violence in the family [31,34-36]. However, findings like these mainly refer to physical violence while the issue of psychological abuse as a single entity is not much researched. This study indicates a clear association between low SES and physical violence, in that regardless of how SES is measured (educational level or income), low SES in the husband is associated with a higher risk of physical violence. However, regarding the association between husbands SES and psychological abuse, the pattern is less clear. Low professional status of the husband is associated with a high risk of this type of violence while the associations with husband's education and household income are weak. The reasons for this pattern might be complex and involving factors like education as both a source of information and a change agent for social norms, which could interact with factors like concordance in spouses' levels of education.

Staying with a co-wife was the strongest risk factor for lifetime physical/sexual violence but interestingly this factor was not associated with psychological abuse in our study. Similar findings was reported in studies from China [28] and Uganda [37].

The suggested difference in socio-demographic risk factors as pertains to physical/sexual and psychological abuse as single entity deserves some attention. A common violence escalation pattern has been described that starts with milder forms of psychological abuse that over time steps up into controlling behaviour and later into serious forms of physical violence [38]. However, we found that a considerable proportion of the participating women had never been physically victimised but psychologically abused and possibly over a longer period of time as the majority (175 women, 20 percent) were 30 years of age or above. This speaks in favour of our hypothesis that the two forms of violence, physical/sexual and psychological abuse alone, occur as separate entities where the perpetrators might differ in several aspects. There is support for this in our findings.

## Conclusion

This study clearly indicates that married women in rural Vietnam are heavily exposed to all forms of serious abuse repeatedly over time from their husbands or male partners. This poses a serious threat not only to the women's health but also to their children and other family members and constitutes a serious violation of women's rights. There is an urgent need for effective support, counselling and treatment of violence victims where health care staff has a role to play as they are to observe professional secrecy and are relatively easy to access. Screening procedures for early detection is of utmost importance and health care staff at all levels are in need of training in how to address victimised women and their partners. Staff at health centres and health posts needs counselling skills and training in how to pose sensitive questions on violence to feel confident in handling complicated situations. Hospital based staff could form a point of referral for treatment and counselling. Locally elected representatives organised in women's unions, people's committees, youth unions and local reconciliation groups could form support groups for counselling. Media has a role to play in creating a debate on this topic and the national level need to be strict on rules and regulations to be updated and followed. Explicit criminalisation of marital rape would send out an important signal on the unacceptability of the use of force within the family.

It could be that the commonly employed way of describing forms of violence (physical, psychological, sexual) could be taken a step further. Overlaps between the different forms are commonly found in studies, but pure psychological abuse seems also to be rather common. It might be that different strategies are needed to reduce and prevent the different forms and combinations of violence, depending on the risk factor pattern and the perpetrators' characteristics.

## Competing interests

The author(s) declare that they have no competing interests.

## Authors' contributions

All three authors are responsible for the design of the study, main responsible for the data collection was NDV, further NDV made all the statistical analysis, the interpretation of findings were made by all three authors, NDV and GK are main responsible for drafting the ms. All authors read and approved the final content of the manuscript.

## Pre-publication history

The pre-publication history for this paper can be accessed here:


